# Epineural stimulation on distal brachial plexus for functional restoration of the upper limb in a primate study

**DOI:** 10.3389/fneur.2025.1515986

**Published:** 2025-03-18

**Authors:** Tianfang Yan, Benjamin C. Fortune, Lingjun Liu, Yan Liu, Taro Kaiju, Takafumi Suzuki, Masayuki Hirata

**Affiliations:** ^1^Department of Neurological Diagnosis and Restoration, Graduate School of Medicine, Osaka University, Suita, Japan; ^2^Center for Information and Neural Networks (CiNet), National Institute of Information and Communications Technology, and Osaka University, Osaka, Japan

**Keywords:** functional electrical stimulation, peripheral nerve, brachial plexus, epineural, cuff electrodes

## Abstract

Restoring upper limb function is critical in individuals with central paralysis, and hand control is a priority in patients with neurological impairments. Functional electrical stimulation with implantable electrodes targeting the peripheral nervous system has the potential to selectively recruit hand muscles and generate multiple functional hand movements. However, the implantation of electrodes in the forearm or elbow areas requires multiple incisions for surgery, and elbow joint movements cannot be performed. In this study, we designed and implanted two epineural cuffs on the median and radial nerves in the distal brachial plexus of a single Japanese macaque (*Macaca fuscata*) monkey. The cuffs were successfully placed via an axillary approach using a single incision. Electrical stimuli were applied to innervate the contraction patterns of the hand, forearm, and triceps muscles relevant to the median and radial nerves. The evoked potentials of the target muscles electrically stimulated the distal brachial plexus to reliably and selectively innervate the upper limb muscles at the functional group level. Our results demonstrated that the distal brachial plexus can be a useful stimulation site for upper limb muscle contraction and that the axillary approach enables electrode placement to peripheral nerves required for upper limb control.

## 1 Introduction

Functional electrical stimulation (FES) is a treatment that involves using mild electrical pulses to muscles or nerves to assist in restoring upper and lower extremity function in individuals who have been paralyzed due to injury to the central nervous system (CNS) (1). Upper limb and hand functionality are particularly important for performing daily activities and increasing the quality of life of individuals with neurological impairments (2). Previous studies have used surface or implantable FES systems in motor neural prostheses to control the arms and hands (3, 4).

Surface FES is more commonly applied clinically during rehabilitation because it is non-invasive. Clinical studies have shown the effectiveness of using surface FES for improving stroke rehabilitation to regain upper limb motor functions (5–7). Innovative technologies, such as flexible multiple-electrode array, have been integrated to generate more selective stimulation (8). However, surface electrodes are applied to the skin and thus have some limitations: they are only suitable for primary muscles close to the skin, have limited ability to selectively innervate individual muscles, require larger currents, and have reverse recruitment effects leading to fatigue (9). Invasive procedures can be more reliable methods to improve selectivity and performance.

Implantable FES targeting branches of the peripheral nervous system is an alternative strategy for muscle recruitment. Research groups have focused on the median, radial, and ulnar nerves to produce selective functional upper-limb movements. Recent studies have demonstrated the potential of a microfabricated transverse intrafascicular multichannel electrode to selectively recruit extrinsic and intrinsic hand muscles, generating multiple functional grips and hand openings in three monkeys (9). The intrafascicular electrodes are positioned within the nerve fascicles near the efferent axons of different muscles (10, 11) by penetrating both the epineurium and the perineurium. However, needle insertion is associated with increased risks of damage and inflammation. The epineural cuff electrode is another type of multi-contact peripheral nerve interface that has been implemented in clinical neuroprostheses aimed at restoring hand function after paralysis (12, 13). Electrodes are placed on the surface of the nerve to avoid fascicle tissue damage. Precise stimulation can be delivered to a small portion of fascicles, thus enable selective muscle activation.

The brachial plexus is a network of nerve fibers that travels through the posterior triangle of the neck into the axilla. Its anatomical structure allows access to multiple branches of the peripheral nerves and innervates muscles from a single proximal location, reduces the placement of multiple electrodes, and simplifies the surgical procedure (14). In a previous study, we verified the potential for surgical access to the peripheral nerves of the upper extremities using a single surgical approach (15). We successfully implanted epineural cuffs on four peripheral nerves at the brachial plexus level that controlled the upper limbs: the median, radial, ulnar, and musculocutaneous nerves.

In this study, we implanted epineural multi-contact FES cuffs on two peripheral nerves at the proximal level just distal to the brachial plexus and aimed to evaluate their ability to selectively innervate the target muscles of the upper extremity. Approaching all five nerves (median, radial, ulnar, musculocutaneous, and axillary) is possible using a single small axillary skin incision to innervate the entire upper extremity. However, in this study, we focused on stimulating the median and radial nerves because these two nerves innervate the major flexor and extensor muscles of the forearm and hand. Recruitment curves were obtained to quantify the selectivity of the various stimulation parameters for multiple contacts.

## 2 Materials and methods

### 2.1 Non-human primate animal model

In this study, we used a single adult male Japanese macaque (*Macaca fuscata*) weighing 10.2 kg. *Macaca fuscata* has an anatomical structure similar to that of humans. The monkey was healthy and exhibited no signs of neurological impairment. The experimental protocol was approved by the Animal Experiment Committee of the Graduate School of Medicine (Approval Number 04-025-000) and the Graduate School of Frontier Biosciences (FBS-22-003-1), Osaka University. All experimental protocols followed the animal research guidelines of the Graduate School of Medicine and Graduate School of Frontier Biosciences, Osaka University, and the NIH guidelines for animal care.

### 2.2 Epineural cuff

**Figure 1:**
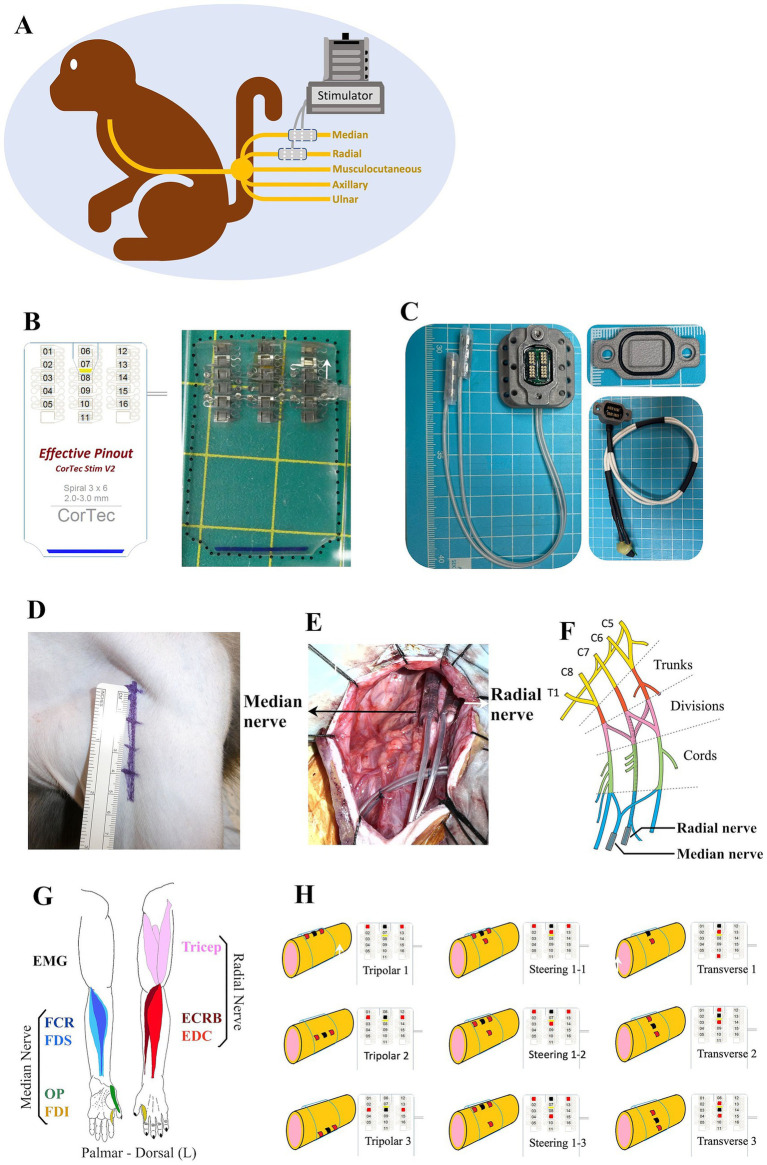
Summary of the method employed in this study. **(A)** Monkey with two epineural cuffs implanted on the median and radial nerves ready for stimulation test. **(B)** Schematic practical graph of the custom Pt/Ir 90/10 spiral nerve cuff (CorTec GmbH, Freiburg, Germany), consisting of 3 × 6 electrodes. **(C)** Percutaneous connecter with a sealing cap and a recording cap. **(D)** 5.5 cm skin incision of axillary approach to access the brachial plexus. **(E)** Intraoperative photograph of the implantation of the cuffs. **(F)** Anatomical structure graph showing the position of implanted cuffs. **(G)** Representation of the flexor and extensor muscles of the monkey upper limb innervated by the median and radial nerve. **(H)** Different stimulation configurations showing the positions of cathodes (red spots) and anodes (black spots).

An epineural cuff was developed to validate the ability of the epineural cuff electrodes to innervate the target muscles of the upper limbs. To reduce current spreading and potentially increase the selectivity of the evoked muscles (16, 17), we used a tripolar stimulus configuration. Before confirming the epineural cuff specifications, a neuroanatomical analysis was performed on two cadaver monkeys. This process provides insights into the required diameter of the nerve cuff. A Pt/Ir 90/10 spiral nerve cuff with 3 × 6 contacts, a self-adaptive diameter of 3 mm, and a length of 30 mm was developed by CorTec (GmbH, Freiburg, Germany) (Figure 1B). We also designed a percutaneous connector as an interface between the implanted cuffs and the stimulator (Figure 1C).

### 2.3 Surgical procedures

Surgery was performed aseptically under general anesthesia with isoflurane (1–3%, inhalation) and continuous monitoring of the monkey’s condition and vital signs. After anesthesia, the monkey was positioned supine on the operation table. A 5.5 cm linear skin incision was made from the coracoid towards the humerus on the left upper limb (Figure 1D). Bleeding was carefully stopped using a bipolar coagulator. The deltopectoral groove was blunted to show the brachial plexus, where the nerve fibers could be observed through the translucent connective tissue. The most proximal portions of the median and radial nerves distal to the brachial plexus were freed from the surrounding tissues. Intraoperative electrophysiological stimulation was used to identify the target nerves. Epineural cuffs were gently implanted into the median and radial nerves (Figures 1E,F).

Acute EMG electrode pairs (NE-215B, Nihon Kohden, Japan) were inserted into each target muscle belly, permitting a bipolar electrode configuration with an inter-electrode distance of 10 mm. We chose four flexor muscles in the hand and forearm predominantly innervated by the median nerve and three extensors innervated by the radial nerve (Figure 1G), where finger flexion is produced by the flexor digitorum superficialis (FDS), wrist flexion is achieved by flexor carpi radialis (FCR), finger opposition is performed with first dorsal interosseous (FDI) and opponens pollicis (OP); finger extension is produced by extensor digitorum communis (EDC), wrist extension is achieved by extensor carpi radialis brevis (ECRB), and elbow extension is performed with triceps. A single suture was placed at the entry location on the skin to secure the electrode fixation. Electrical stimulation was used to assist in identifying the target muscles. In addition, a single reference electrode was implanted proximal to the left clavicle. After the experiment, the monkey was awakened and placed in an observation cage under free-moving conditions. Carprofen (3 mg/kg, subcutaneous injection) was used as an analgesic, and ceftriaxone sodium (25 mg/kg, intramuscular injection) was used as an antibiotic.

### 2.4 Electrophysiology

Electrical stimuli were applied to the epineural cuffs of the monkey under general anesthesia (Figure 1A). During stimulation, we simultaneously recorded compound muscle action potentials (CMAPs) from the seven target muscles (Table 1 and Figure 1G). All stimulations and EMG recordings were performed using a processor unit (RZ2; Tucker-Davis Technologies, United States) and a Subject Interface (SIM10-6, Tucker-Davis Technologies, United States) with Synapse software. Disposable subdermal needle electrodes were connected to a needle electrode adapter (S-BOX16; Tucker-Davis Technologies, United States). EMG activities were collected at a sampling rate of 12,207 Hz and filtered using a 20–500 Hz order bandpass filter.

**Table 1:** Muscles for EMG recoding, their functions, and the nerve supply.

Muscles (EMG)	Functions	Nerve supply
Flexor carpi radialis	Wrist flexion	Median N.
Flexor digitorum superficialis	Finger flexion	
First dorsal interosseous	Index opposition	
Opponens pollicis	Thumb opposition	
Extensor digitorum communis	Finger extension	Radial N.
Extensor carpi radialis brevis	Wrist extension	
Triceps	Forearm extension	

Electrical stimuli were administered as constant-current charge-balanced cathodic-first biphasic pulses. We used a 32-channel ZIF-Clip headstage (ZC32-P, Tucker-Davis Technologies, United States) to deliver pulses with a pulse width of 150 μs and a frequency of 1 Hz. Across different experimental conditions, we varied the amplitude from 100 μA to 4,000 μA and performed six repetitions of each current step. We employed different stimulation configurations: the traditional longitudinal tripolar, tripolar with steering, and transverse tripolar (Figure 1H). In traditional tripolar stimulation, we utilized five cathodes, whereas in transverse tripolar stimulation, all six cathodes were used. To mitigate the effects of fatigue, we enforced a 1-min rest period between changes in the stimulation cathodes.

### 2.5 Muscle recruitment and selectivity

Muscle recruitment curves were generated by recording the CMAPs obtained from each target muscle. Data within a 50-ms data window were captured for each stimulation pulse, spanning 10 ms before and 40 ms after the onset of stimulation. This process was set up using Tucker-Davis Technologies Synapse software and performed in real time.

We used the peak-to-peak amplitude of the evoked CMAPs to investigate the relationship between the evoked muscle response and stimulation intensity. The maximum CMAP amplitude recorded for each muscle during the experiment was used to normalize the CMAPs for each muscle separately, thus enabling the production of normalized recruitment curves for each muscle. The selectivity of each muscle was assessed by calculating the selectivity index (SI). SI was calculated using the equation outlined by Badi et al. (9) as follows.


$${SI}_{m}={\mathrm{CMAP}}_{m}-\frac{\sum_{n\neq m}^{M}{\mathrm{CMAP}}_{n}}{M-1}$$

where *m* is the muscle of interest, *M* is the total number of muscles, and CMAP is the normalized CMAP. The SI calculation ranges from −1 to 1, where −1 indicates full activation of all non-target muscles and zero activation of the muscles of interest, and 1 indicates full activation of the target muscle and zero activation of all non-target muscles. All the calculations were performed using MATLAB (MathWorks, 2023a).

### 2.6 Histological evaluation

Nerve samples from the distal brachial plexus were dissected after transcardial perfusion and embedded in paraffin for histological evaluation. Choline acetyltransferase (ChAT) is used as a marker of cholinergic neurons and specifically labels motor neurons (18). The coronal sections were processed for immunohistochemical staining with Nissl and anti-ChAT antibodies.

The tissues first underwent antigen retrieval treatment for 10 min in sodium citrate buffer (0.1 M citric acid, 0.1 M sodium citrate, pH 6.0) at 121°C. After inactivation of endogenous peroxidase with 3% H_2_O_2_ in methanol for 15 min at room temperature, the tissues were incubated with primary rabbit anti-ChAT antibody (1:250, bs-0042R, Bioss, United States) overnight at 4°C. After washing with phosphate buffer saline (0.05 M PBS, pH 7.6), the tissue was labeled with peroxidase secondary antibody (424144, Histofine Simple Stain MAX PO; Nichirei, Japan) for 30 min at room temperature. The sections were visualized using 3,3′-diaminobenzidine tetrahydrochloride (Nichirei) at room temperature. Sections were counterstained with Mayer’s hematoxylin and examined under a microscope. Images of the sections were captured using a microscope (BZ-X800, Keyence, Japan) at 4× and 40× magnification, manually outlined, and quantitatively measured using BZ-X800 software.

## 3 Results

### 3.1 Electrodes implantation

**Figure 2:**
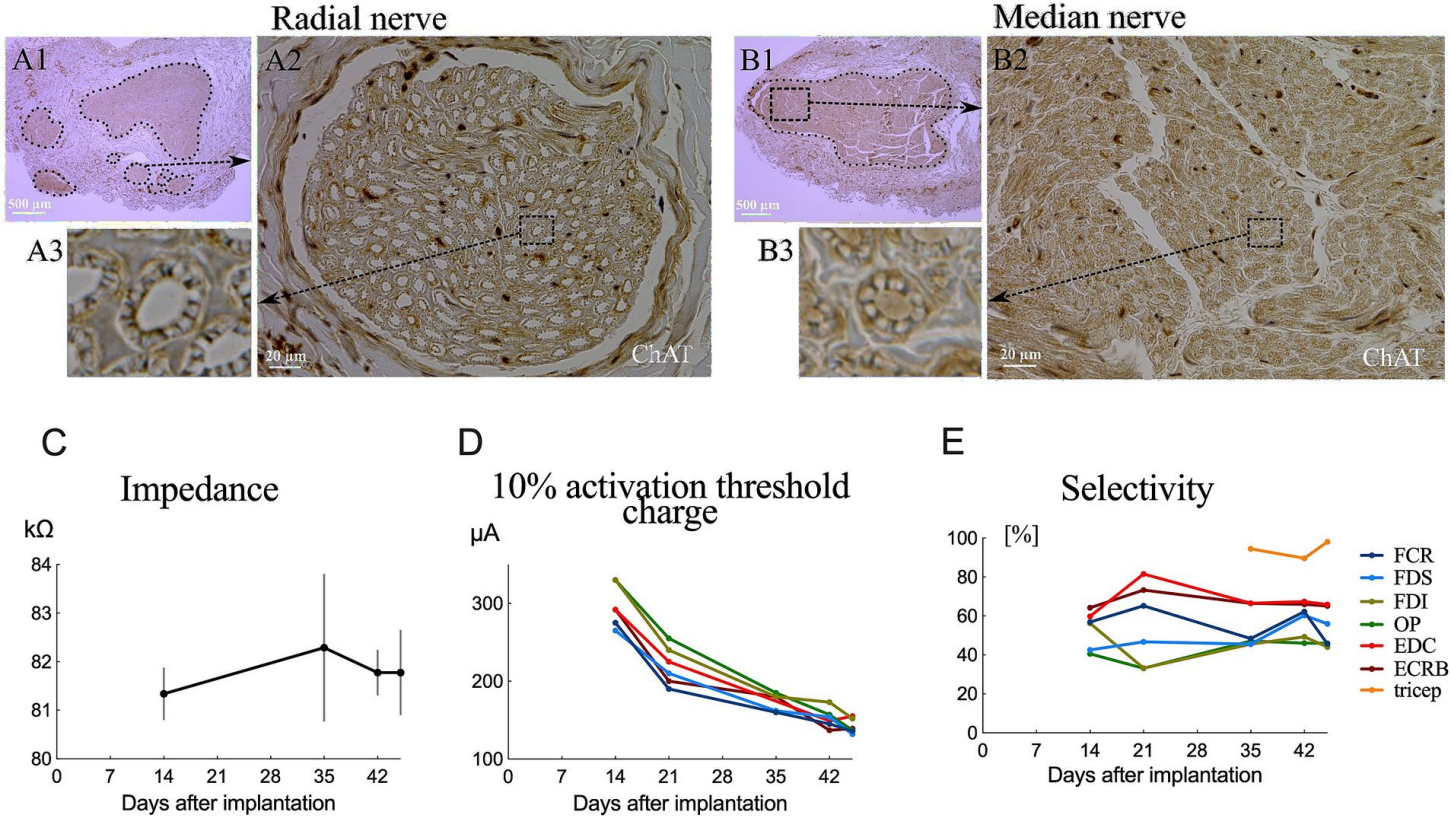
Fascicular morphology of the median **(B1)** and radial **(A1)** nerves at the distal portion of the brachial plexus in monkeys. Cross section of ChAT-labeled median **(B2,B3)** and radial **(A2,A3)** nerve showing the mapping of the fascicles (outlined by dotted lines). **(C)** Impedance changes of the averaged results over all electrodes 45 days after implantation. **(D)** Chronic evaluations of the activation charge threshold (10% recruitment). **(E)** Change in selectivity index of tripolar stimulation over 45 days after implantation.

We successfully implanted two cuff electrodes onto the most distal portion of the median and radial nerves just distal to the brachial plexus through the axillary approach with a 5.5 cm skin incision (Figures 1D–F). This approach is applicable and efficient for placing epineural cuffs on up to four nerves that control the entire upper extremity. However, the axillary nerve lies behind the axillary artery and is occluded by the teres major, making it difficult to access (15). Additionally, we investigated the cross-sectional area of fascicles in the implanted area. Our results showed a single large fascicle in the median nerve and one larger-diameter fascicle accompanied by several smaller-diameter fascicles in the radial nerve (Figures 2A,B) harvested from the implanted site (Figure 1F). Immunohistochemical results of ChAT staining revealed a heterogeneous distribution of motor neurons within each fascicle of the median and radial nerves (Figures 2A,B). Chronic electrode impedance measurements demonstrated that the impedance was relatively stable (Figure 2C). The 10% activation threshold of recruitment gradually decreased 1 month after implantation, indicating a stable microenvironment between the electrodes and the nerve surface (Figure 2D). The selectivity index of longitudinal tripolar stimulation over 45 days after implantation (Figure 2E) showed a relatively stable performance of the implants.

### 3.2 Muscle recruitment and selectivity

**Figure 3:**
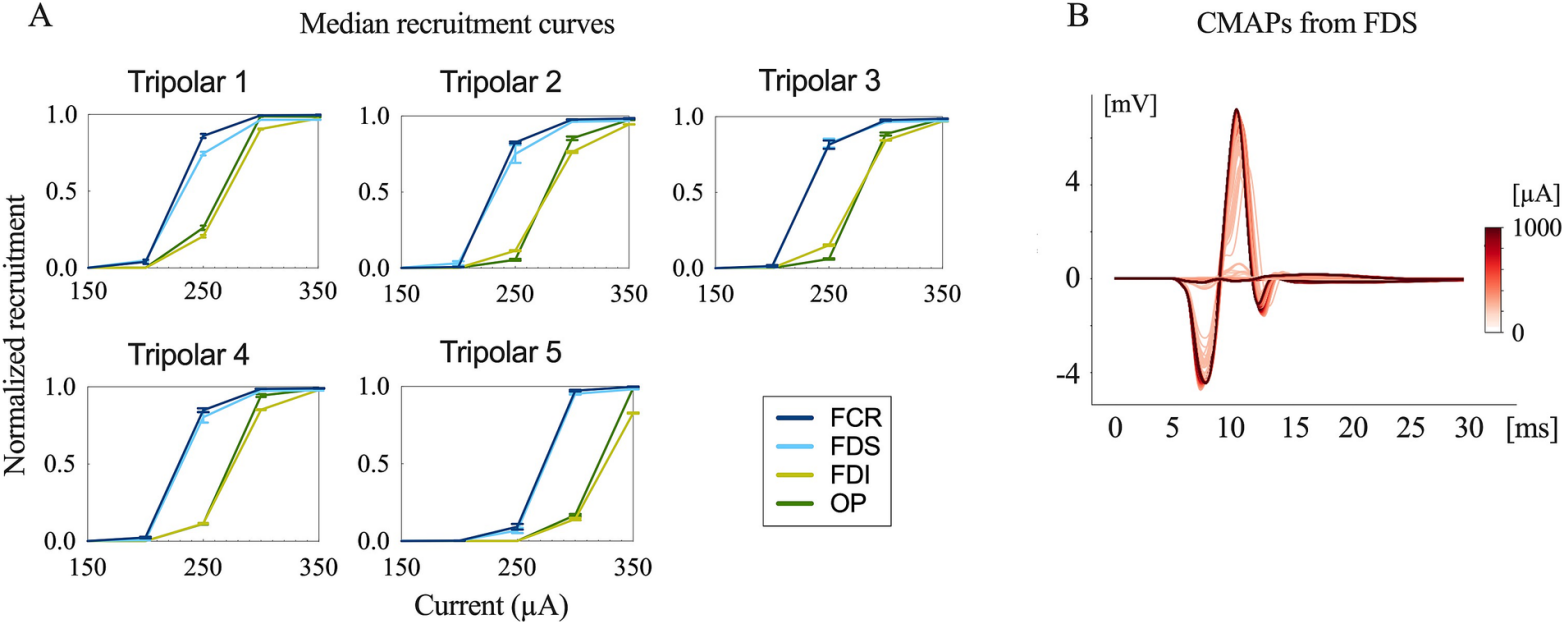
Evoked compound muscle action potentials and recruitment curves. **(A)** Recruitment curves for the five longitudinal tripolar configurations obtained during median nerve stimulation. The *x*-axis has been scaled to focus on the transition phase of the subthreshold and saturation region. **(B)** Evoked compound muscle action potentials for the different stimulation amplitudes recorded from FDS (flexor digitorum superficialis) (stimulation onset at 0 ms).

**Figure 4:**
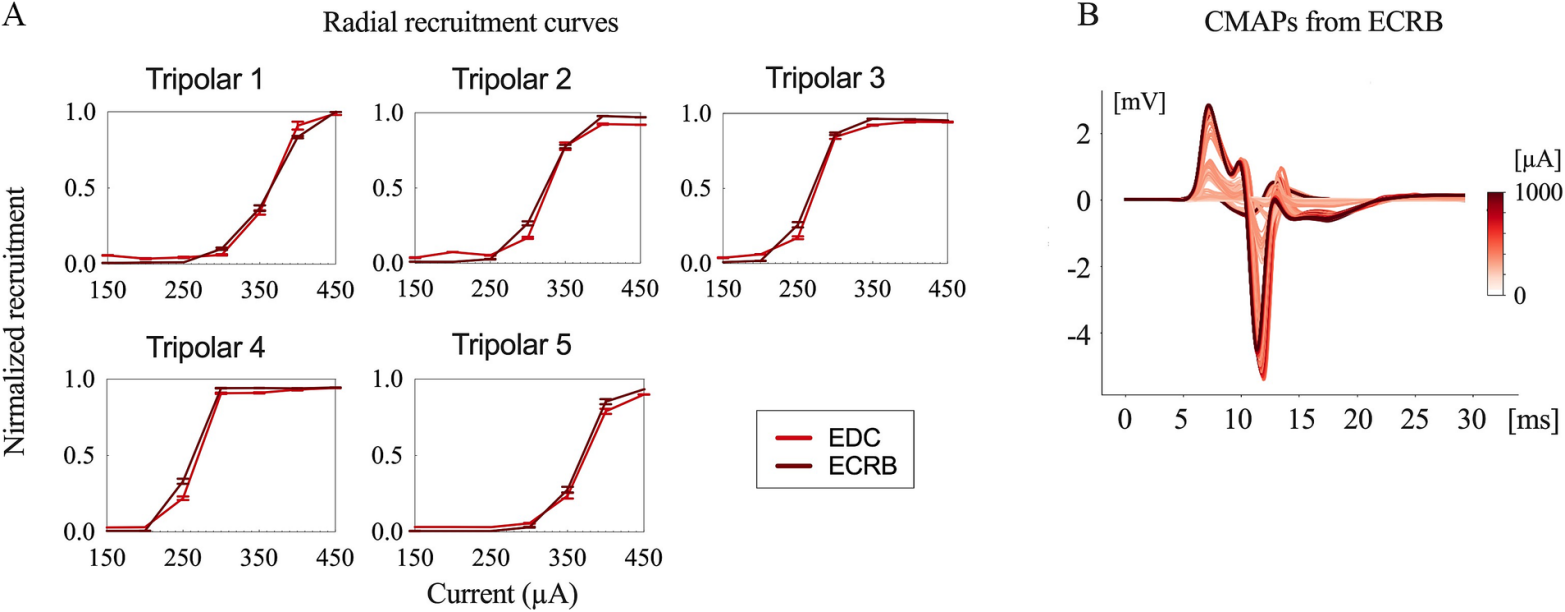
Evoked compound muscle action potentials and recruitment curves. **(A)** Recruitment curves for the five longitudinal tripolar configurations obtained during radial nerve stimulation. **(B)** Evoked compound muscle action potentials for the different stimulation amplitudes recorded from ECRB (extensor carpi radialis brevis).

Typical contraction patterns of muscles relevant to the innervated peripheral nerves were observed following electrical stimulation of the epineural cuffs. The recorded CMAPs validated that the electrical stimuli on the median and radial nerves innervated all the recorded upper limb muscles. The peak-to-peak response increased until saturation as the amplitude of the stimuli increased from the subthreshold to saturation (Figures 3, 4).

The time delay between stimulus onset and the recorded CMAPs was approximately 6 ms for both the median and radial nerves. This indicates that forearm muscle recruitment was achieved through the direct innervation of motor axons in macaque monkeys (9).

The recruitment curves showed normalized activation of the upper limb muscles during the median (Figure 3) and radial (Figure 4) nerve stimulation in all five tripolar sets. To visually clarify the recruitment order, we focused on the region of the recruitment curves corresponding to the range between the subthreshold and saturation responses. Our results revealed that specificity FDI and OP when stimulating the median nerve, whereas the triceps exhibited an earlier order than ECRB and EDC when stimulating the radial nerve. These results suggest that the proximal muscle groups have lower thresholds than the distal muscle groups when stimulating peripheral nerves at the distal portion of the brachial plexus.

**Figure 5:**
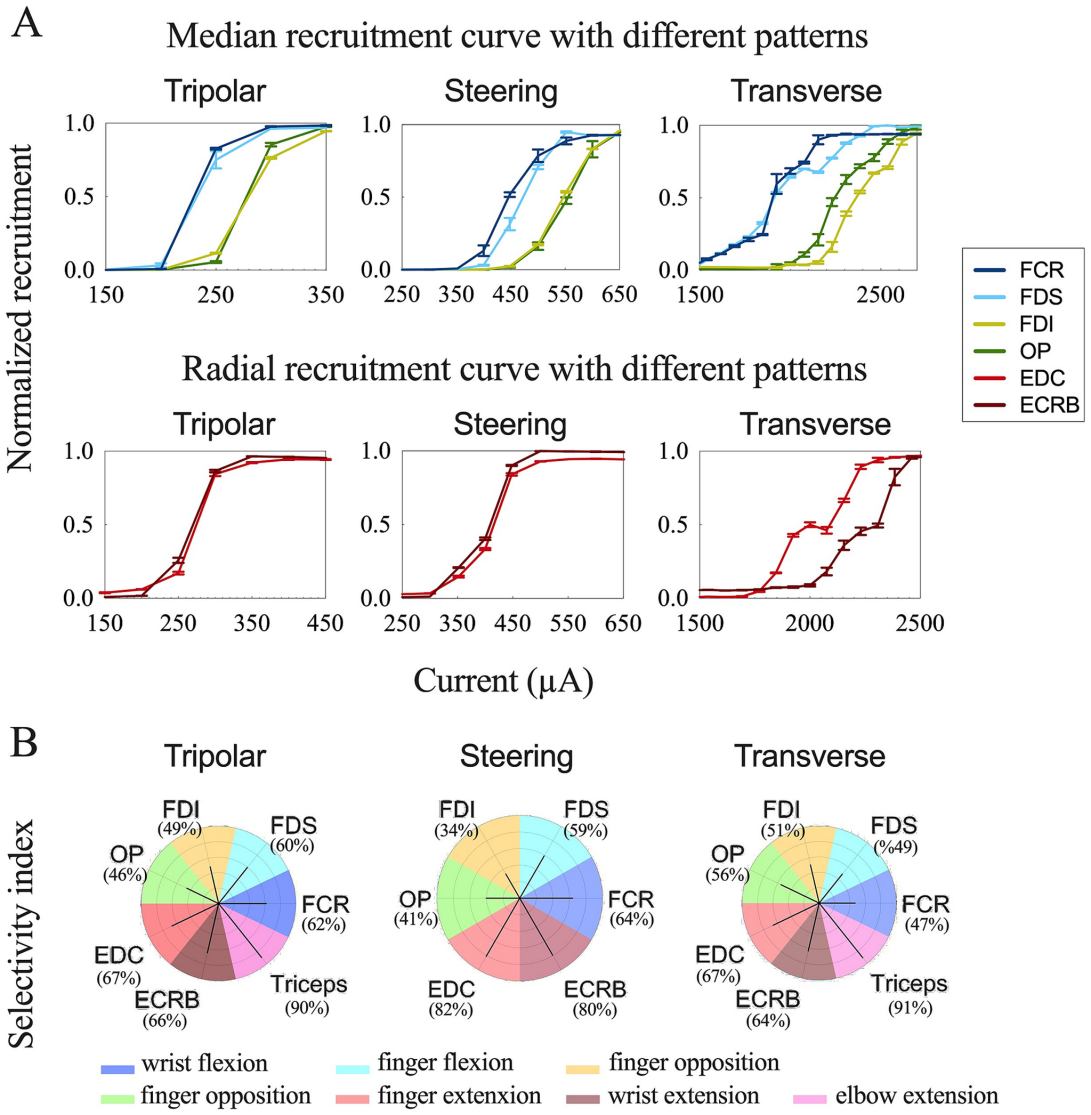
Comparison results of different stimulation configurations. **(A)** Recruitment curves for longitudinal tripolar, tripolar with steering, and transverse tripolar stimulating on median (top) and radial (bottom) nerves. **(B)** Summary of selective muscle activation for the median and radial nerves. Selectivity for each muscle was achieved using different stimulation configurations. The dark line represents the selectivity index. The color of each polar plot separates each muscle.

**Figure 6:**
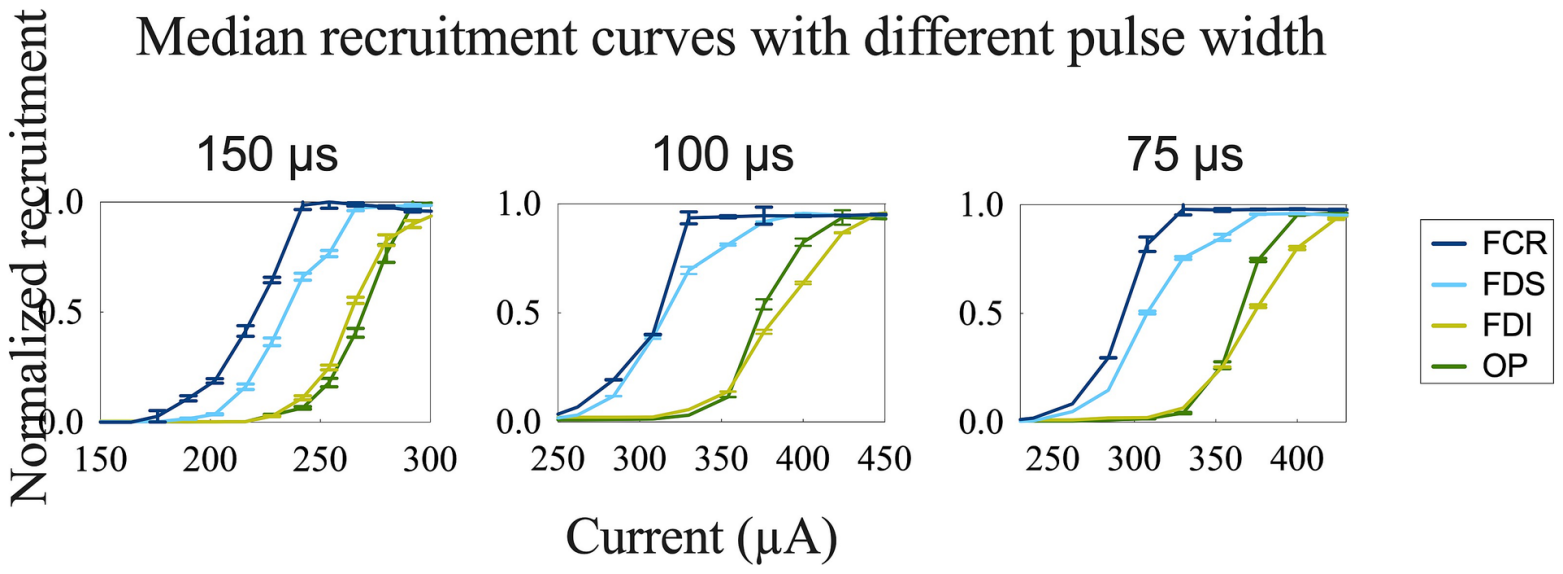
Recruitment curves for comparison of different stimulation pulse widths obtained during longitudinal stimulation on the median nerve.

To achieve more selective innervation of the muscles within the same group, we tested different stimulation parameters by changing the configuration and pulse width. Transverse tripolar and steering configurations increased the subthreshold, saturation, and span compared to the longitudinal tripolar configuration and could distinguish the two recruitment curves of muscles from the same group (Figure 5A). More complete figures are provided in the Supplementary Figures 1, 2. The stimulation with a shorter pulse width required a higher amplitude to reach the recruitment threshold (Figure 6). However, the effect of modifying the stimulation configuration and pulse width was limited to the innervation of different muscles in the same group.

Further, we quantified the selectivity of muscle contraction by the three types of tripolar stimulation using SI, a metric that reflects the normalized activation of each muscle with respect to non-target muscles. The maximum selectivity indices for each muscle and the corresponding configuration are outlined in Table 2 and Figure 5B.

**Table 2:** Maximum selectivity index for the upper arm muscles and the corresponding stimulation protocol.

Muscle	Selectivity index	Configuration	Amplitude (μA)
Longitudinal tripolar stimulation			
FCR	62.19%	Tripolar-3	160
FDS	60.26%	Tripolar-5	192
FDI	49.32%	Tripolar-1	434
OP	46.01%	Tripolar-3	480
ECRB	67.08%	Tripolar-1	2,500
EDC	65.96%	Tripolar-1	1,528
Triceps	89.64%	Tripolar-2	708
Transverse tripolar stimulation			
FCR	46.15%	Transverse-6	2,050
FDS	48.6%	Transverse-3	2,250
FDI	51.35%	Transverse-3	1,950
OP	55.62%	Transverse-3	2,000
ECRB	64.34%	Transverse-5	1,350
EDC	66.86%	Transverse-5	1,850
Triceps	91.31%	Transverse-1	950
Tripolar with steering stimulation			
FCR	64.34%	Steering 5-6	550
FDS	59.49%	Steering 4-5	450
FDI	34.22%	Steering 5-4	1,000
OP	40.94%	Steering 5-3	800
ECRB	79.89%	Steering 5-2	800
EDC	81.65%	Steering 3-1	500

## 4 Discussion

We implanted two customized epineural cuff electrodes onto the most proximal portion of the median and radial nerves just distal to the brachial plexus in a macaque monkey using the axillary approach with a single 5.5 cm skin incision. Electrical stimulation at the distal brachial plexus level demonstrated the ability to selectively innervate the upper limb muscles at the functional group level. Our results highlight the feasibility of distal brachial plexus stimulation for control of the entire upper extremity.

In this study, we validated that the distal brachial plexus can be a useful stimulation site for FES aimed at controlling all upper limb muscles. Previous studies have implanted electrodes on peripheral upper limb nerves at different sites (9), usually with median electrodes placed near the elbow and radial electrodes near the epicondyle. However, this strategy requires longer cables between each electrode and connector, complicating the surgical procedure. Moreover, the biceps (musculocutaneous nerve) and triceps (radial nerve) cannot be innervated by stimulating the elbow. One feasible approach to access the musculocutaneous nerve is the axillary approach, which targets the distal branch of the brachial plexus.

The brachial plexus is a network of nerve fibers connecting the cervical roots and the motor and sensory nerves of the upper extremities. This anatomical feature makes it easy to access and place the epineural cuffs on all five peripheral nerves, innervating the upper extremities through a single axillary approach. This highlights the feasibility of producing entire upper-extremity muscle control, including the triceps, which is the principal muscle responsible for elbow extension. The axillary approach is the most popular method of accessing the brachial plexus. Only one small skin incision is required for this approach, thus decreasing the surgical site and simplifying the cabling system of the multi-cuff implantable device.

Epineural recruitment at the distal portion of the brachial plexus was found to engage the intrinsic muscles of the hand and arm and selectively activate a large number of flexor and extensor muscles. Fascicular topography of the arm nerves has shown that, in the fascicles, the deep middle fibers innervate distal muscles and that superficial fibers innervate proximal muscles (19). Our results revealed that lower amplitude stimulation can innervate proximal muscles, and as the stimulus amplitude increases, deeper fibers are activated, allowing more distal hand muscles to be innervated. Additionally, the stimulation sites of the cathode did not affect the recruitment selectivity. However, we did not differentially stimulate each muscle in the same functional group (for example, the FCR and FDS both innervate wrist flexion). A cross-sectional examination of the motor fiber distribution at the distal brachial plexus showed one main large fascicle with fewer subfascicles, indicating that it is difficult to selectively match the targeted muscles and specific area of the fibers of the terminal branches. We attempted three different stimulation configurations (longitudinal tripolar, tripolar with steering, and transverse tripolar) with different cathode sites to selectively activate deep fibers; however, the effect was not satisfactory.

Intraneural transverse intrafascicular recruitment at the elbow level produces fine hand movements (9). However, stimulation of the radial nerve at the epicondylar segment cannot activate the triceps, and elbow joint extension cannot be achieved. Our results showed higher maximum selectivity values obtained through epineural stimulation than those obtained using intrafascicular stimulation (9) (Table 2). This may be attributed to the relatively high selective recruitment of epineural stimulation on motor fibers lying near the nerve surface and the relatively weak recruitment on the fibers deep inside the nerve. Additionally, owing to the design of the intrafascicular electrodes, only monopolar stimulation can be applied. A tripolar configuration can achieve better control of the stimulation electric field than a monopolar electrode (20).

Persistent motor impairment in the arm after damage to the CNS due to injury or disease, such as in post-stroke patients and those with incomplete spinal cord injuries, can make patients functionally dependent on others for daily activities. Epineural FES may be a useful tool for inducing the contraction of paralyzed muscles and performing basic joint activities. The functional movement control of the upper limbs in complete paraplegics and tetraplegics remains promising. Stimulation has the advantage of functioning and exercising over a long period (21). Implantable electrodes are placed perineurally to deliver smaller currents on a small portion of fascicles, thus enable more selective activations to target muscles and reduce the occurrence of fatigue (22). Our epineural cuffs were designed to accommodate multiple nerve diameters and not harm the peripheral nerve, thereby extending stable implantation time. Previous anatomical research on the upper limb muscles and nerves in monkeys and humans (19, 23) has revealed that the structure of the arm is preserved in primates, suggesting that our approach in monkeys could be translated to humans.

Sensory fibers were also founded in each fascicle in both median and radial nerves with a heterogeneous distribution (Figures 2A,B). Unwanted activation on afferent sensory fibers can contribute to the evoked muscle contraction by the synaptic recruitment of motor neurons in the spinal cord (H-reflex) (24, 25). However, sensory axons have a longer strength-duration time constant and lower rheobase than motor axons and are more preferentially recruited by wider pulses widths (0.5–1 ms) and lower stimulating amplitudes (26, 27). Our stimulating pulse widths were limited shorter (75 μs, 100 μs, 150 μs), and preferentially activated motor fibers. CMAPs results from EMG recording (Figures 3B, 4B) showed a short onset latency of 5 ms, indicating that the muscular responses were mediated by a direct peripheral pathway and not by transsynaptic Ia-mediated reflex responses (28).

This study has certain limitations. In this early-stage study, we implanted only two cuffs on the radial and median nerves to simplify the experiment. We did not use pharmacological nerve blocks to inhibit the effects of the spinal circuits. The acute EMG electrodes were inserted at slightly different points on each experimental day, resulting in some variance in the selectivity analyses. In future experiments, a chronic EMG recording method is recommended to minimize this influence.

In the next step, epineural stimulation with a higher frequency rather than a single pulse is required to generate functional joint movements. In addition, cuffs were placed on all four nerves (median, radial, ulnar, and musculocutaneous) to test functional movements and tasks. The surgical approach also requires further optimization to reliably access the axillary nerve. In terms of the stimulation method, more stimulus patterns and parameters must be tested to improve the selectivity of the evoked muscles. Fully implantable devices with wireless charging coils and rechargeable batteries are another significant improvement for the long-term implantation of electrodes. For a long-term research plan, the FES effect can be improved by combining it with brain-computer interface technology for motor control. We expect that this study will develop brain-controlled peripheral nerve FES interface research to support upper limb functionality and further benefit humans with neurological impairments.

## 5 Conclusion

The distal brachial plexus may be a useful stimulation site for functional electrical stimulation using an axillary approach with a single skin incision. Electrical stimulation at the distal brachial plexus level demonstrated the ability to selectively innervate the upper limb muscles at the functional group level. The stimulation sites on the cathode did not affect the recruitment selectivity. Our results highlight the feasibility of distal brachial plexus stimulation for control of the entire upper extremity.

## Data Availability

The original contributions presented in the study are included in the article/Supplementary material, further inquiries can be directed to the corresponding author.
