# Supplementary material for: Epineural stimulation on distal brachial plexus for functional restoration of the upper limb in a primate study
Source: Front Neurol. 2025 Mar 18;16:1515986. doi: 10.3389/fneur.2025.1515986 (PMC11958176; doi:10.3389/fneur.2025.1515986)
Supplement: Supplementary file 1 [file Table_1.docx]

Supplementary Material

#
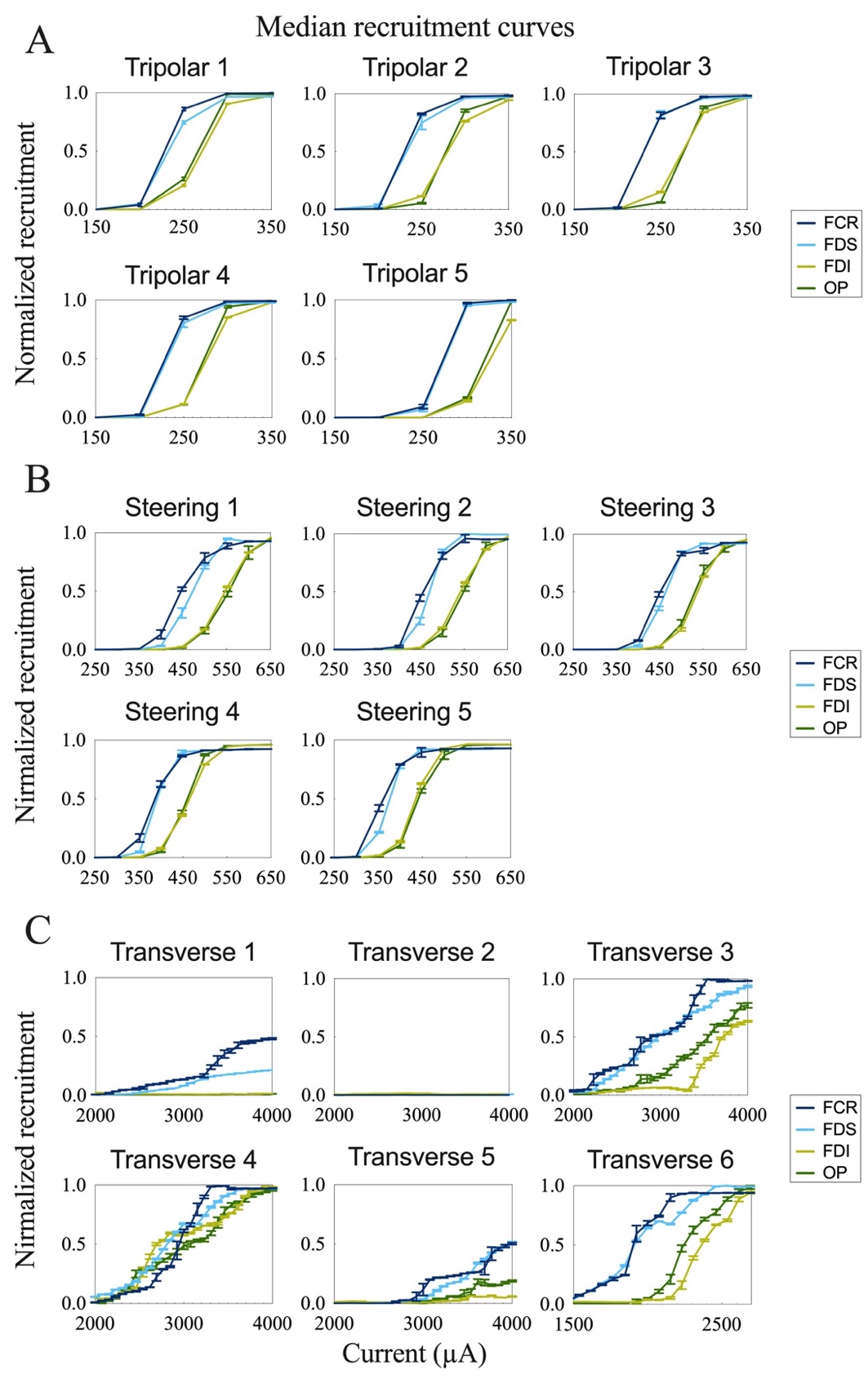


# Supplementary Figure 1. Recruitment curves for different stimulation patterns. A: five longitudinal tripolar configurations; B: tripolar with five steering configurations; and C: six transverse tripolar configurations stimulating obtained during median nerve stimulation.

**
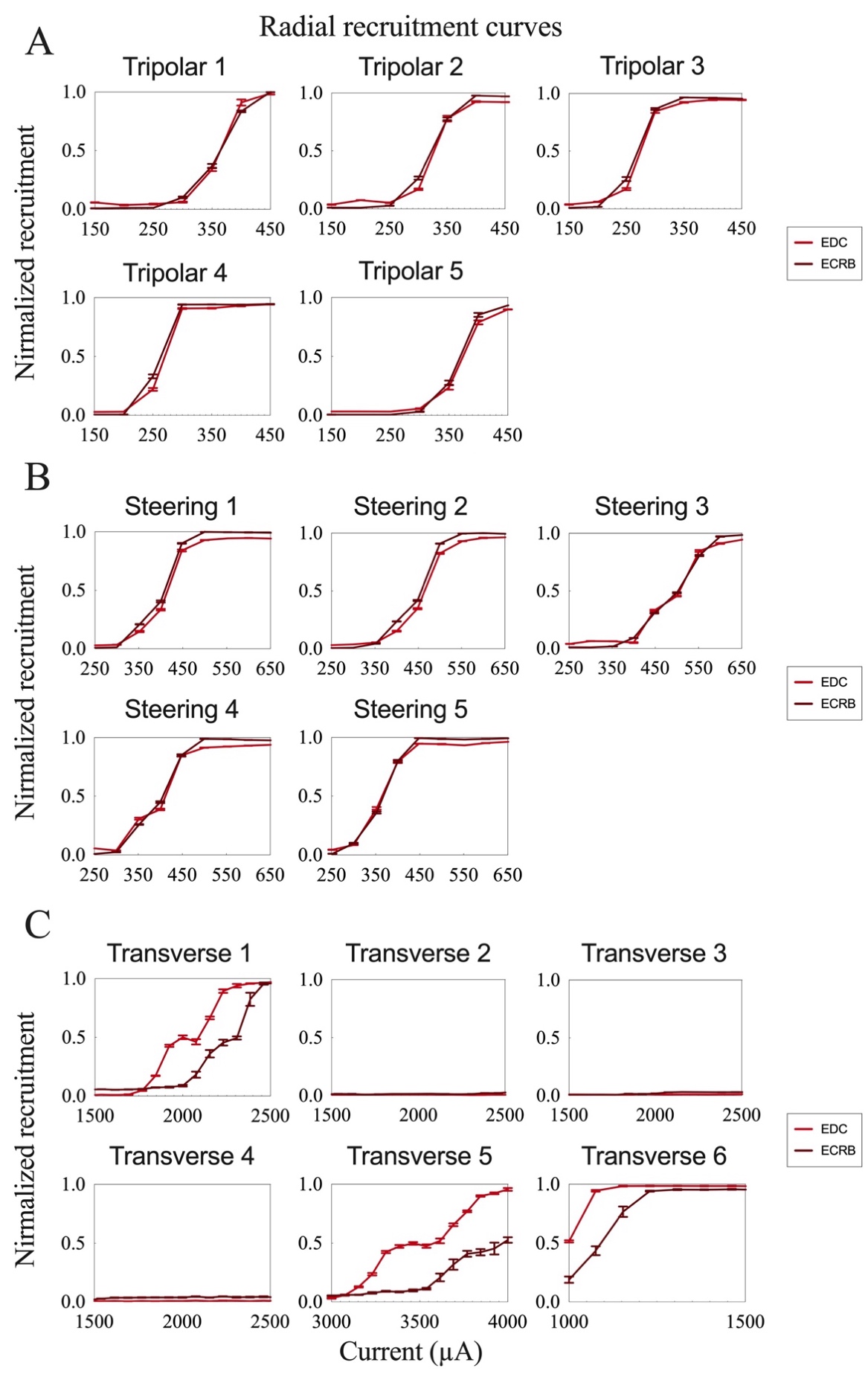
**

**Supplementary Figure 2.** Recruitment curves for different stimulation patterns. A: five longitudinal tripolar configurations; B: tripolar with five steering configurations; and C: six transverse tripolar configurations stimulating obtained during radial nerve stimulation.
